# Five EMT-Related Gene Signatures Predict Acute Myeloid Leukemia Patient Outcome

**DOI:** 10.1155/2022/7826393

**Published:** 2022-10-07

**Authors:** Jing Qi, Jiawei Yan, Muhammad Idrees, Saeedah Musaed Almutairi, Rabab Ahmed Rasheed, Usama Ahmed Hussein, Mostafa A. Abdel-Maksoud, Ran Wang, Jun Huang, Chen Huang, Nana Wang, Dongping Huang, Yuan Hui, Chen Li

**Affiliations:** ^1^Department of Hematology, The First Affiliated Hospital of Wannan Medical College, Wuhu, Anhui 241001, China; ^2^Primary and Secondary Health Care Department, Lahore, Pakistan; ^3^Department of Botany and Microbiology, College of Science, King Saud University, Riyadh 11451, Saudi Arabia; ^4^Histology & Cell Biology Department, Faculty of Medicine, King Salman International University, South Sinai, Egypt; ^5^Anatomy Department, Faculty of Medicine, King Salman International University, South Sinai, Egypt; ^6^Public Health Center, The First Affiliated Hospital of Xi'an Jiaotong University, Xi'an, Shanxi, China; ^7^Department of Biology, Chemistry, Pharmacy, Free University of Berlin, Berlin 14195, Germany

## Abstract

**Background:**

The epithelial mesenchymal transition (EMT) gene has been shown to be significantly associated with the prognosis of solid tumors; however, there is a lack of models for the EMT gene to predict the prognosis of AML patients.

**Methods:**

First, we downloaded clinical data and raw transcriptome sequencing data from the TCGA database of acute myeloid leukemia (AML) patients. All currently confirmed EMT-related genes were obtained from the dbEMT 2.0 database, and 30% of the TCGA data were randomly selected as the test set. Univariate Cox regression analysis, random forest, and lasso regression were used to optimize the number of genes for model construction, and multivariate Cox regression was used for model construction. Area under the ROC curve was used to assess the efficacy of the model application, and the internal validation set was used to assess the stability of the model.

**Results:**

A total of 173 AML samples were downloaded, and a total of 1184 EMT-related genes were downloaded. The results of univariate batch Cox regression analysis suggested that 212 genes were associated with patient prognosis, random forest and lasso regression yielded 18 and 8 prognosis-related EMT genes, respectively, and the results of multifactorial COX regression model suggested that 5 genes, CBR1, HS3ST3B1, LIMA1, MIR573, and PTP4A3, were considered as independent risk factors affecting patient prognosis. The model ROC results suggested that the area under the curve was 0.868 and the internal validation results showed that the area under the curve was 0.815.

**Conclusion:**

During this study, we constructed a signature model of five EMT-related genes to predict overall survival in patients with AML; it will provide a useful tool for clinical decision making.

## 1. Introduction

Acute myeloid leukemia (AML) is the most common type of acute leukemia in adults, characterized by a low remission rate, high relapse rate, high disease-specific mortality, and poor prognosis. The incidence of AML increases with age, and more than 20,000 cases are diagnosed per year in the United States, and over 50% of patients died from this disease [1, 2]. Although advances in immunology, cytogenetics, and molecular biology have laid the groundwork for stratified and precise treatment of AML, up to 50% of patients with normal karyotype have a wide range of clinical outcomes [3]. Thus, it is crucial to develop more risk standards and predictive models for predicting the prognosis and directing treatments of AML.

AML is a highly heterogeneous group of diseases with uncontrolled proliferation and differentiation of abnormally clonal myeloid stem cells. The application of next-generation sequencing (NGS) technology and bioinformatic analysis has provided systemically studies of genome and transcriptome data to unravel the mutational spectrum, epigenetic landscape, and RNA interaction network of these clonal leukemia cells [4], which help to construct different models to predict prognosis and discover potential biomarkers of AML [5, 6]. Epithelial to mesenchymal transition (EMT) is a dynamic process with the transition of epithelial cells to mesenchymal cell phenotype, which has played important roles in embryonic development and wound healing, and this process is also thought to be involved in cancer progression and therapy resistance [7, 8]. The overexpression of EMT markers and EMT transcription factors (TFs) has been proved to correlate with tumor aggressiveness and poor prognosis [9, 10]. In addition, recent studies have shown that cancer cells with the EMT process may contribute to immune escape and drug resistance, thereby reducing the effect of immunotherapy and chemotherapy [11–13]. As in hematological malignancies, previous studies already indicated a correlation between some EMT markers and poor prognosis. For example, the upregulation of vimentin, one of the EMT markers, was found associated with poor clinical outcome in AML patients [14], and downregulation of ZEB1 in AML cells can inhibit the invasive ability [15]. Taken together, all these indicate that EMT markers and EMT-TFs involve in the progression of AML, and EMT-related signatures could be used as potential target for predicting prognosis. However, more of its specific biological function still needs to be explored.

## 2. Materials and Methods

### 2.1. Data Acquisition and Preprocessing

A total of 173 AML samples were obtained from the The Cancer Genome Atlas (TCGA) database, a landmark cancer genomic program, which contains more than 20,000 primary cancer and matched normal samples spanning 33 cancer types. The corresponding transcriptome sequencing data of the AML dataset were downloaded and normalized to FPKM format. EMT-related genes were obtained from the dbEMT2.0 database, which contains a total of 1184 experimentally confirmed EMT-related genes. Then, we extracted the expression profiles of EMT-related genes from the normalized matrix based on the obtained EMT-related gene names. Finally, the expression profiles were combined with clinical information to generate a new matrix, and 30% of the data were randomly extracted from this matrix and set as the test set. For clinical data, it is necessary that the enrolled patients have a complete follow-up time, those samples with missing survival time and survival status are excluded from the cohort, and overall patient survival is defined as the endpoint event.

### 2.2. Batch Univariate COX Regression Screening for Prognosis-Associated EMT Genes

Not all EMT-associated genes affect patient survival; therefore, further screening of EMT-associated genes that affect patient prognosis is necessary. We included 1184 EMT-related genes from the EMT database in a univariate COX regression model with *p* < 0.05 as a filtering condition in order to screen for risk factors that affect the prognosis of AML patients.

### 2.3. Machine Learning to Screen Prognosis-Associated EMT Genes

Randomized survival forest and lasso regression are machine learning algorithms that are often used for dimensionality reduction analysis. The prognostic genes obtained from the above analyses were included in the random survival forest, which was performed by the R package “random forest”, and the importance threshold of the variables was set to 0.45. Variables above this threshold were included in the lasso regression for further dimensionality reduction.

### 2.4. Multivariate Cox Regression and Model Construction

We first included the prognostic factors obtained from the lasso regression into the multivariate Cox regression to screen the independent risk factors affecting the prognosis of AML patients and then constructed a multigene prognostic model based on the coefficients of the regression model.

### 2.5. Model Efficacy Assessment and Internal Validation

We assessed whether there was a difference in the prognosis of patients in the high- and low-risk groups using the log rank test and then assessed the applied efficacy of the model using the area under the ROC curve. In addition, to validate the stability of the model, 30% of the randomly selected data from the original data were used as the test set for this evaluation.

## 3. Results

### 3.1. Training Set Prognosis-Related EMT Gene Screening and Model Construction

**Figure 1:**
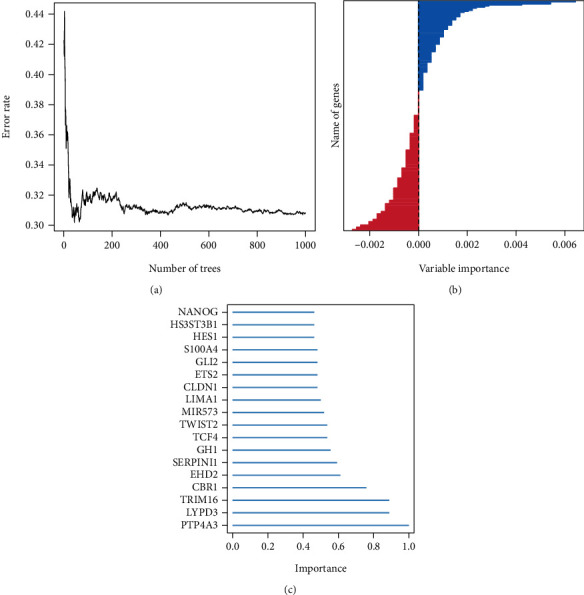
Random survival forest select candidate EMT-related prognosis genes. The error estimate probability (**a**), the bar plot of genes (**b**), and candidate important genes (importance >0.45) (**c**).

**Figure 2:**
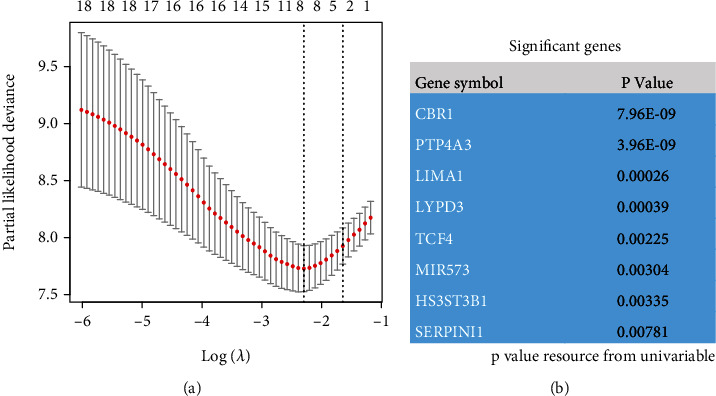
Lasso regression model select candidate EMT-related prognosis genes. Lambda takes the minimum value; a total of eight candidate genes are selected (**a**), and (**b**) demonstrates the prognostic value of these eight genes.

The results of the univariate batch COX regression analysis suggested that 212 EMT-related genes were associated with prognosis in AML patients. Top 20 prognosis-related genes are presented in Table 1. These 212 genes were included in the random survival forest model, and a total of 18 prognosis genes were selected when the gene importance was set greater than 0.45 (Figures 1(a)–1(c)), and these 18 genes were subsequently included in the lasso regression model for dimensionality reduction analysis, and a total of 8 genes were selected (Figures 2(a) and 2(b)). Further, we included these 5 genes into the multifactorial COX regression model, and a total of 5 genes were selected, and they were considered as independent risk factors affecting the prognosis of patients (Table 2). These 5 genes were CBR1, HS3ST3B1, LIMA1, MIR573, and PTP4A3. Five EMT-associated genes were further modeled for signature based on COX regression coefficients.

### 3.2. Performance of EMT-Associated Signature

**Figure 3:**
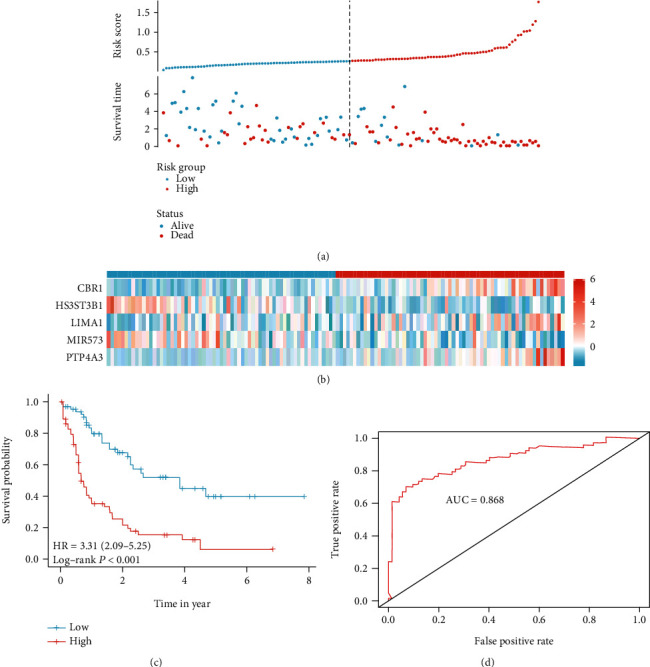
Construct model in training data set, based on the Cox regulation model, a five EMT-related gene signature was constructed: the risk score and the survival status distribution (**a**) and the heat map of five genes in high- and low-risk group (**b**). The survival curve show high-risk score patients with a worse outcome, compared with low-risk score patients (**c**). The area under the receiver operating characteristic of model was 0.868 (**d**).

**Figure 4:**
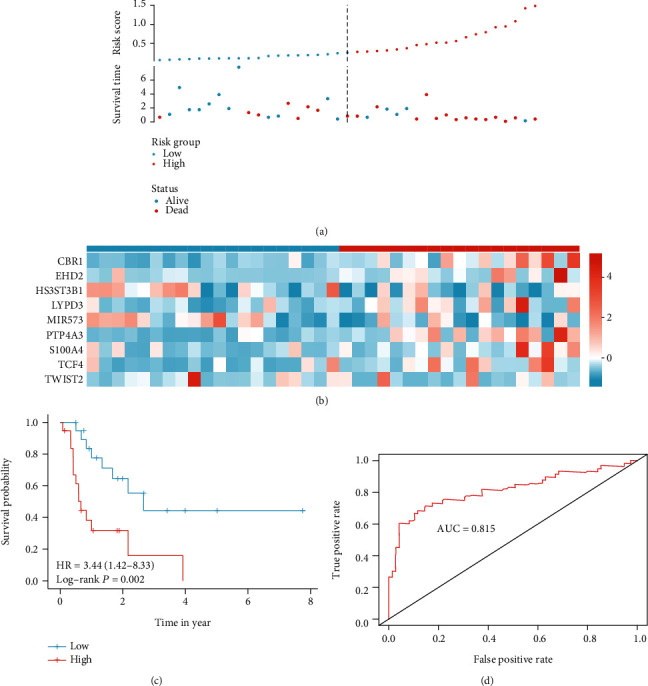
Validate model in test data set, a five EMT-related gene signature was validated: the risk score and the survival status distribution (**a**) and the heat map of five genes in high- and low-risk group (**b**). The survival curve show high-risk score patients with a worse outcome, compared with low-risk score patients (**c**). The area under the receiver operating characteristic of model was 0.815 (**d**).

We first calculated the risk score for each patient based on this model. To evaluate the performance of the signature model, patients were divided into high and low groups according to the median value of risk score expression, and the results suggested that the disease-specific survival rate of high-risk patients was significantly lower than that of low-risk patients, and the comparison between groups was statistically different (*p* < 0.001) (Figures 3(a)–3(c)), and the ROC results suggested that the predictive efficacy of the model was likewise. The area under the curve was 0.868 (Figure 3(d)). In addition, to verify the stability of the model, 30% of the total sample was selected for the internal validation of the test set. The results suggested that the same between-group survival differences existed in the test set (Figures 4(a)–4(c)). In addition, the results suggest that the model has strong stability with an area under the ROC curve of 0.815 (Figure 4(d)). This result suggests that the model has a strong stability.

## 4. Discussion

AML is a deadly and highly heterogeneous disease due to extensive genomic changes and molecular mutations, which have been incorporated in the updated 2017 European LeukemiaNet (ELN) risk stratification guidelines [16]. Breakthroughs in NGS technology have not only explored the molecular mechanisms of this disease but also bring the AML into the era of small molecule inhibitor therapy. More studies are devoted to exploring new prognostic models based on the genetic and molecular profiling to uncover more potential therapeutic targets [4–6]. In the present study, we constructed a predictive model based on the EMT-related signature to provide a visual predictive tool for AML, which might lay the foundation for exploring the role of EMT in hematological malignancies.

Epithelial cells provide intercellular adhesion by cell-cell cohesion and are essential for maintaining the integrity and barrier function of multicellular structures. However, epithelial cells transform into mesenchymal cells to acquire more complex structures and functions of organs during embryonic development and wound healing, which is termed EMT [17, 18]. The quiescent epithelial cells in adults reactivated and primed for the EMT under various internal and external changes, which facilitate tumor cells to invade the extracellular matrix and evade the immune elimination [19]. The downregulation of the cell adhesion protein E-cadherin and cytoskeletal rearrangements, including downregulation of keratin and upregulation of vimentin, are the main features of EMT, which cause ultimately tumor progression and metastasis. Several EMT-TFs have been well identified to coordinate the process, such as SNAIL/SNAI1, SLUG/SNAI2, and TFs of the TWIST and ZEB families [20]. Given that EMT is associated with tumor invasiveness and metastasis, as well as its molecular properties, some EMT-related signatures have been developed to predict the prognosis of cancers and the response to immunotherapy. A recent study reported an EMT-related gene signature for the prognosis of human bladder cancer [21], and Chae et al. [22] analyzed the immune landscape of NSCLC (nonsmall cell lung cancer) patients based on EMT scores to predict the response of patients to immunotherapy. Although some previous studies have shown the role of EMT makers and EMT-TFs in AML, no EMT signature has been applied to predict the prognosis of AML [14, 15].

As shown in our study, five EMT-related genes (CBR1, HS3ST3B1, LIMA1, MIR573, PTP4A3) were selected by random forest algorithm as the prognostic in TCGA-LAML cohort as a training set. Then, AML patients were divided into high-risk and low-risk groups based on the EMT-related signature risk score. The results demonstrated that patients in the low-risk group have longer OS than in the high-risk group, which were also validated in internal datasets. Carbonyl reductase 1 (CBR1) belongs to the short dehydrogenase (SDR) family, which could promote AML cell resistance to daunorubicin and be a risk gene in AML patients [23]. However, it is still unclear whether CBR1 can lead to progression and drug resistance through EMT in AML. A previous study has shown that heparan sulfate D-glucosamine 3-O-sulfotransferase 3B1 (HS3ST3B1) participates in the biosynthetic steps of heparan sulfate (HS) and positively contributed to acute AML progression by induction of VEGF expression, which also involves in the regulation TGF-beta-mediated EMT in NSCLC [24, 25]. LIMA1 (LIM domain and actin binding 1), also known as epithelial protein lost in neoplasm (EPLIN), has been known to play differential roles in the progression and metastasis of certain cancers [26, 27]. Downregulation or phosphorylation of EPLIN can alter the expression of some EMT elements such as E-cadherin and ZEB1 via Wnt-catenin signaling pathway, thus promotes the EMT process. While the exact mechanism of LIMA1 in AML remains unknown [27]. The role of MIR573 in EMT of tumors is still controversial. Wang et al. [28]. revealed that MIR573 can inhibit TGF*β*1-induced EMT in prostate cancer, while another study indicated MIR573 associated with the EMT in cervical cancer cell growth and metastasis [29]. As so far, the expression of MIR573 has been confirmed in AML cell line (HL-60) and thought as a regulator in responsiveness to inorganic substances [30]. Protein tyrosine phosphatase of regenerating liver 3 (PRL-3), encoded by PTP4A3 gene, has been proved to promote EMT through PI3K/AKT pathway and Src-ERK1/2 pathways in a variety of tumors [31, 32], which is also a hazard factor with poor survival in AML [33]. All these hint the prognostic role of EMT-related gene signature in AML. Furthermore, given that the general condition of the patients is also included in the risk stratification of the disease in addition to the genomic profile [16], a predictive model was constructed based on the EMT-related genes, which demonstrated powerful predictivity.

## 5. Conclusion

During this study, we constructed a signature model of five EMT-related genes to predict overall survival in patients with AML; it will provide a useful tool for clinical decision making. However, our study still has some limitations. First, more datasets need to be included for better validation. Second, further function experiments regarding of the core genes are required to clarify the role of EMT-related genes in AML.

## Figures and Tables

**Table 1:** Top 20 candidate genes of univariate Cox regression analysis results.

Candidate genes	Univariate Cox regression			
	HR	95% CI		*P* value
		Low	High	
PTP4A3	1.021726223	1.014321763	1.029184734	6.96E-09
CBR1	1.03820974	1.025067827	1.051520139	7.96E-09
ROR1	8.179147658	3.372478696	19.83658384	3.33E-06
ETS2	1.005307794	1.00295453	1.00766658	9.55E-06
HIP1	1.014075523	1.007666162	1.020525653	1.56E-05
PLA2G4A	1.021276597	1.011531433	1.031115646	1.68E-05
SRC	1.041105298	1.021883572	1.060688587	2.27E-05
KRT7	2.305624508	1.5494694	3.43079016	3.80E-05
HOXB7	1.017736146	1.008665558	1.026888302	0.000118629
PEBP4	9.238964316	2.976207974	28.68027449	0.000119556
UCP2	1.001228192	1.00059024	1.001866551	0.000160359
CDK5	1.025260006	1.011990418	1.03870359	0.000174577
CCL22	1.521162335	1.219639632	1.897228318	0.000198028
RNF8	1.147867946	1.066800293	1.235096044	0.000223934
LIMA1	1.073789995	1.033493977	1.115657159	0.00026414
SPRR2A	18025860.7	2044.952808	1.58894E+11	0.000312518
BMP2	1.363657752	1.150470944	1.616348917	0.000348845
LYPD3	1.593586359	1.231965357	2.061354624	0.000387329
STIM2	1.04818812	1.021292087	1.075792468	0.000387406
BAG3	1.030482529	1.013356191	1.047898312	0.000445426

**Table 2:** Multivariate Cox regression analysis of candidate genes.

Candidate genes	Multivariate Cox regression				
	Coef	HR	95% CI		*P* value
			Low	High	
CBR1	0.0286	1.0290	1.0147	1.0436	6.62E-05
HS3ST3B1	−0.0458	0.9552	0.9131	0.9993	0.0466
LIMA1	0.0415	1.0423	1.0078	1.0781	0.0160
MIR573	−0.0134	0.9867	0.9716	1.0020	0.0888
PTP4A3	0.0145	1.0146	1.0064	1.0228	0.0004

## Data Availability

All data used in the study were from the publicly available The Cancer Genome Atlas (TCGA) (https://portal.gdc.cancer.gov/).
